# Treatment-related adverse events as a source of financial hardship in young adults with breast cancer: a qualitative study

**DOI:** 10.1007/s00520-025-09887-8

**Published:** 2025-09-26

**Authors:** Sara P. Myers, Ramona G. Olvera, Sandy Lee, Karen Shiu, Tessa Blevins, Willi L. Tarver, William E. Carson, Electra D. Paskett, Samilia Obeng-Gyasi, Ann Scheck McAlearney

**Affiliations:** 1 Division of Surgical Oncology, Department of Surgery, The Ohio State University, Columbus, OH USA; 2 Comprehensive Cancer Center, The Ohio State University, Columbus, OH USA; 3 CATALYST, Center for the Advancement of Team Science, Analytics, and Systems Thinking in Health Services and Implementation Science Research, College of Medicine, The Ohio State University, Columbus, OH USA; 4 University of Cincinatti College of Medicine, Cincinatti, OH USA; 5 Department of Psychology, The Ohio State University, Columbus, OH USA; 6 Division of Cancer Prevention and Control, Department of Internal Medicine, College of Medicine, The Ohio State University, Columbus, OH USA; 7 Department of Family and Community Medicine, College of Medicine, The Ohio State University, Columbus, OH USA

**Keywords:** Breast cancer, Financial toxicity, Young adults, Treatment costs

## Abstract

**Purpose:**

Rates of breast cancer are increasing among young adult (YA) women aged ≤ 40 years. YAs face unique challenges, including being at high risk for financial hardship. Treatment-related adverse events may represent a modifiable and often overlooked source of financial hardship. In this interview-based study, the narratives of YAs with breast cancer were analyzed to understand how treatment-related adverse events contributed to medical and non-medical costs and long-term economic burden.

**Methods:**

In this secondary analysis of semi-structured interviews characterizing financial toxicity among adult women with stage I–IV breast cancer treated at The Ohio State University Comprehensive Cancer Center (OSUCCC) between 1/1/2015 and 12/31/2019, previously transcribed and coded data from women ≤ 40 years old was analyzed using inductive and deductive approaches.

**Results:**

Twenty breast cancer survivors aged ≤ 40 years participated. Treatment-related adverse events emerged as an important factor contributing to financial toxicity. Participants described complications in nearly every organ system, many of which were disabling and required intervention. While indirect (e.g., job loss, reduced work hours) and direct sources (e.g., compression garments for lymphedema) of costs were noted to cause psychological distress and impact treatment adherence, participants did articulate possible solutions for reducing financial hardship (e.g., direct cash transfer, financial navigation).

**Conclusion:**

Treatment-related adverse events can contribute to financial toxicity after breast cancer through direct and indirect costs. Among young adults, indirect costs can include those that result from vocational disruption. Strategies to reduce the risk of financial toxicity should be included in care pathways to address complications of treatment itself.

## Introduction

The increasing prevalence of breast cancer in young adult (YA) women aged ≤ 40 years [1] has motivated interest in issues of survivorship among this demographic. YA breast oncology patients face unique challenges, chief among them being elevated risk for financial hardship [2]. Several factors contribute to this economic vulnerability. Premenopausal women are more likely to present with aggressive tumor subtypes and advanced disease stage [3], both of which may be indications for costly multimodality treatment. Moreover, high deductible health insurance [4], limited financial reserves [5], and lack of support networks may amplify the consequences of medical debt on quality of life and clinical outcomes (i.e., financial toxicity) among YAs [6, 7].

Despite recognition that younger women are at heightened risk for financial toxicity [8], solutions to counteract the negative impact of treatment costs remain elusive. Promising interventions such as financial navigation rarely target YAs [9]. Increasing health insurance literacy so that YAs are aware of coverage for services and financial assistance has also been proposed with encouraging pilot data [10]. These programs, however, are available only while the patient is receiving active treatment. Accordingly, resources to mitigate long-term financial hardship are lacking.

These issues highlight that further investigations are needed to understand modifiable sources of financial toxicity and to design interventions that span not only the acute phase of cancer treatment but also extend into survivorship. Recent data demonstrating indirect and direct costs associated with treatment-related adverse events [11] suggest continued care of treatment-related complications may be a major but intervenable source of financial strain. While there is little data to support whether transient side effects of treatment (e.g., nausea, diarrhea) might also impact long-term economic hardship, we postulate that unanticipated sequelae related to job loss or protracted medical debt could contribute to financial toxicity. As an initial step toward understanding the general importance of both acute and chronic treatment-related complications to financial hardship among this demographic, we analyze the narratives of young adults with breast cancer with the intent of describing what these events might be, whether and how they contribute to overall experience with cancer treatment, and how they affect medical and non-medical costs and long-term economic burden.

## Methods

### Study setting, design, and participants

In this secondary analysis of qualitative data from semi-structured interviews aimed at characterizing financial toxicity among adult women with stage I–IV breast cancer treated at The Ohio State University Comprehensive Cancer Center (OSUCCC) between 1/1/2015 and 12/31/2019 [12], we focus on treatment-related adverse events in the subset of participants aged ≤ 40 (i.e., YAs). As the impact of treatment-related adverse events on financial hardship has been underevaluated, this analysis considers both short-term transient complications and those that lead to chronic morbidity. Participants were eligible for inclusion if they received at least one of their oncologic treatment modalities (e.g., surgery, chemotherapy, radiation) at the OSUCCC. As the original investigation was designed to interrogate experiences of women at high risk for financial toxicity, participants were required to meet at least one of the following criteria: age ≤ 40 at time of breast cancer diagnosis, self-identifying as Black or African American given existing data showing risk of financial hardship among racially minoritized groups who face inequality [13], residing in a rural county as defined by the 2013 USDA Rural–Urban Continuum Codes, or classified as low income on the basis of having Medicaid coverage for ≥ 1 billing encounter [14, 15]. While the original analysis of participants’ narratives described short and long-term financial hardship for all subgroups defined as being at high-risk for financial toxicity, this secondary analysis specifically highlights financial consequences of treatment-related adverse events, a theme that had not previously been considered, in the YA cohort. After obtaining institutional review board approval for this study, purposive sampling was used to recruit patients in the aforementioned subgroups via telephone and email. Verbal consent was obtained prior to conducting interviews. A $50 gift card was provided as an incentive to participate in the initial study.

### Data collection and analysis

One-on-one interviews and one focus group were conducted virtually over video-conferencing by trained researchers between September 2021 and March 2022. The semi-structured interview guide interrogated the following domains: treatment experience, medical and non-medical costs, indirect costs, and barriers to resources and support. Audio recordings of the interviews were transcribed and patient identifiers were redacted. Sociodemographic and clinical data were abstracted from the electronic medical record and kept separately from transcribed data with a project-created identifier linking the secured datasets. After creating and agreeing upon a codebook for thematic analysis of narratives using the Financial Toxicity Framework created by Witte et al. [16], three investigators independently reviewed and coded the entire dataset. This secondary analysis was restricted to codes that described women’s experience with breast cancer treatment, and specifically, those that were classified under the subdomain of complications during or after treatment. Two investigators independently reviewed codes to summarize emergent themes. The reporting of our methods, data, and analysis is in accordance with the COREQ criteria for qualitative research.

## Results

Of the 50 women that participated in the original study, 20 (40%) were ≤ 40 years old at the time of their breast cancer diagnosis and included in our analysis. Table 1 summarizes patient, disease, and treatment characteristics for this subset. Several treatment-related adverse events resulting in both direct costs and indirect costs were identified. Participants elaborated on facilitators and barriers to addressing financial hardship.

**Table 1:** Patient, tumor, and treatment characteristics

Characteristic	*n* (%); *N* = 20
***Sociodemographics***	
Age (years), median (IQR)	35 (20, 39)
Race/ethnicity	
Non-Hispanic Black	3 (15%)
Non-Hispanic White	17 (85%)
Distance to cancer center (miles), median (IQR)	17 (13.3, 73.1)
Insurance status at time of interview*	
Private	15 (75%)
Medicaid	4 (20%)
Medicare	1 (5%)
***Tumor characteristics***	
Clinical stage at diagnosis	
0/I	8 (40%)
II	8 (40%)
III	2 (10%)
IV	2 (10%)
Tumor molecular subtype	
HR +	12 (60%)
HR +/HER2 +	4 (20%)
HER2 +	1 (5%)
HR −/HER2 −	3 (15%)
***Treatment characteristics***	
Systemic therapy (chemotherapy, immunotherapy, targeted treatment)	11 (55%)
Surgery	18 (90%)
Radiation	13 (65%)
Endocrine therapy	15 (75%)

*Insurance categories for the participating patient cohort were mutually exclusive

### Types of treatment-related adverse events

Patients described a wide range of complications and toxicities affecting nearly every organ system (Table 2). These included breast cancer-related lymphedema (BCRL), cognitive decline, autonomic dysfunction, and psychological distress as well as toxicities that affected cardiopulmonary, hematologic, reproductive, ophthalmologic, mucocutaneous, and aerodigestive systems. Descriptions of these experiences reference all three categories of adverse events as described by the National Cancer Institute’s Common Terminology Criteria for Adverse Event (CTCAE) lexicon: (1) laboratory-detected abnormalities, (2) observable events based on physical exam, and (3) symptomatic adverse events [17]. While degree of severity was not always readily apparent from narratives, some women did describe events that warranted grade 3/4 classification based on the CTCAE. These included supplemental oxygen for a pulmonary embolus, change in medication secondary to cardiac toxicity, a pneumothorax requiring thoracostomy tube, and marrow stimulating factors for neutropenia.

**Table 2:** Representative quotes describing categories of treatment-related adverse events based on the National Cancer Institute’s Common Terminology Criteria for Adverse Events (CTCAE)

CTCAE category	Representative quote
Laboratory-detected	*My white blood cells…kept dropping* *I spent nearly a week in the hospital. And then when I came home, it was another week or so of oxygen because my levels were dropping so much*
Measurable by physical exam	*I ended up having heart issues due to that, the chemo. I went into a flutter, RVR, and I had an ablation* *Like my arms, even for like lymphedema, the top of my arms. Its like my waist is a medium to large, but the tops of my arms are at least a 1X*
Primarily symptomatic with or without observable features	*I was so sick from chemo. I was throwing up; I had massive diarrhea* *When they say chemobrain is a thing- it absolutely is a thing. And I’m not near as sharp as I used to be. And it’s, you know….My quick recall and it’s just, you know, it’s just not there; I just don’t remember a lot of things the way that I used to*

### Direct costs

Participants contextualized complications by reporting direct costs to pay for treatments of the complication itself or its sequela (Table 3). Specifically, some women endorsed treatment nonadherence as a result of cost with statements such as.

*I have to wear knee braces every day…they cost $400. I know that the prescription probably ran out, is probably outdated, because its been sitting there since I dropped it off. I don’t have the $400 to go get brand-new knee braces.*

**Table 3:** Representative quotes describing direct costs resulting from treatment-related adverse events

Treatment-related adverse event	Representative quote
Breast cancer-related lymphedema	*I had some complications with lymphedema and the sleeve and the glove that I needed to wear was not, I think one set was covered. But if I wanted multiple sets, that I had to pay for, which seems silly, because one set is not going to work. You have to launder those things and you need a spare because I would have one that I’d exercise in*
Pain	*And then I had some side effects from the Tamoxifen that I took after I had my daughter and I wanted acupuncture, and it wasn’t covered*
Gonadotoxic medications	*Fertility is huge. I guess depending on what your sort of healthcare situation is. The costs can definitely add up*
Hematologic toxicities	*Was it my hemoglobin? No, my white blood cells, I think, kept dropping and they were like we can give you this shot. I think it started with an “N.” It was kind of like there were things that I knew were really, really expensive*
Ophthalmic toxicities	*I have to get special contacts that are really expensive because my eyes are so dry because now I’m in artificial menopause. So that’s a lot of money. And I don’t have eye insurance*
Oropharyngeal toxicities	*I don’t have dental insurance. The extra teeth exams because one of the chemos is really hard on your teeth*

One woman reported that the cost of surgeries to address implant rupture after her mastectomy with reconstruction was influencing her decision about whether to decline further esthetic procedures:

*I’m actually now facing another surgery because one of my implants is ruptured…I am seriously considering taking the implants out and leaving them out because of the constant up-keep, constant appointments. The possibility of this continuing to happen…I still have $30,000 already, that’s going to add to it…. So the financial cost is weighing on my decision now more than the aesthetic side of things was back then.*

### Indirect costs

All references to indirect costs involved ability to return to work or job performance. As with direct costs, indirect costs influenced the decision to pursue treatment for complications. One woman who was experiencing lymphedema reported declining physical therapy because of the burden of taking time off of work for frequent appointments (Table 4). Neurocognitive symptoms such as “brain fog” and fatigue were factors in declining work performance and consideration for long-term disability.

**Table 4:** Representative quotes describing indirect costs resulting from treatment-related adverse events

Treatment-related adverse event	Representative quote
Breast cancer-related lymphedema	*I’m actually not going to physical therapy now, not because of co-pay, but because taking time off work and the drive up there*
Cognitive dysfunction	*And I couldn’t work because of brain fog and disassociation and all kinds of stuff*
Fatigue	*I mean, I guess the only thing that would have been helpful was not having to be as stressed about the insurance during that month that everything was happening. And if I was at the same school district and my long-term policy for disability wouldn’t have been there, I probably would have taken a little bit more time before I went back in the classroom. Because the exhaustion was really, really hard, those first couple months back in the classroom, because teaching is just not a normal job*
Pulmonary embolism	*I had a pulmonary embolism, they believe from Tamoxifen. And so I then, so again, that was more time off work*
GI toxicities	*I was so sick from the chemo. I was throwing up, I had massive diarrhea. It was horrible. So… my supervisor made me work from home for the two months leading up to my first surgery, my double mastectomy…I was out of my office for four months*

### Facilitators and barriers to addressing financial hardship

As women described their experiences with treatment-related events, some alluded to resources that either would have been helpful or that were effective in alleviating financial burden. One participant who suffered from tamoxifen-associated bone pain reported that she didn’t “know what agency or insurance company would be able to help [her] get [her braces] so [she could] afford them.” Another woman shared this sentiment, emphasizing the importance of financial navigation:

*She pointed out as many resources as she could, as far as grants and different things that were available to people who need financial assistance. So, you know, I had those options for sure. The appointment with endocrinology, they did at no cost….*

Another woman with hematologic toxicities who said she her treatment involved expensive shots offered: “They did all the due diligence to make sure that I knew, for the most part, what my responsibility would be.”

Even when resources were available, being overwhelmed by having to navigate cancer treatments while considering expense served as a barrier to utilizing resources:

*You’re not quick to seek out those resources, you’re so overwhelmed. Which are the ones? Which is a good one? Then there’s this whole pride piece where people don’t like to ask for help, which is ridiculous.*

Direct assistance in the form of cash transfers and grants was helpful for covering costs of care and minimizing burden on caregivers/loved ones. A participant reported that the grant she received from a charitable organization “covered some of [her] medicine,” so that she felt “well taken care of…. [She] wasn’t thinking [her] family was going to have to declare bankruptcy or anything.” Financial support from family was also reported as effective for offsetting costs with one woman expressing gratitude that her “grandparents paid for one of [her] wigs and that was really helpful.”

## Discussion

In this secondary analysis of semi-structured interviews with breast cancer survivors aged ≤ 40 years, treatment-related adverse events emerged as an important factor contributing to financial toxicity. Participants described complications in multiple organ systems, some of which resolved, others of which were disabling and required intervention. While indirect and direct sources of costs were noted to cause psychological distress and impact treatment adherence, participants did articulate possible solutions for reducing financial hardship.

Although adverse events from oncology treatment have been shown to impact health-related quality of life [18], most evidence focuses on the interference with activities of daily living [19] with little consideration for patients’ long-term financial wellbeing. Moreover, few studies have elucidated how short-term adverse events that resolve after active treatment (e.g., chemotherapy-related gastrointestinal toxicities) might have unanticipated sustained effects that contribute to financial hardship. As oncology providers have moved towards implementing value-based care, patient-reported outcomes have become a priority [20]. Given the economic burden of cancer care, financial toxicity is a major impediment to delivery of high-quality care [21]. Our study provides concrete examples illustrating both how short-term and chronic adverse events can contribute to costs incurred and how economizing behaviors are used to offset additional costs. For instance, women who experienced arthritides as a result of their treatment discussed being unable to afford symptom relief due to the prohibitive costs of acupuncture and supportive braces. One woman reported that complications after her implant-based reconstruction caused such severe financial hardship that she was compelled to reconsider her post-mastectomy preferences. As another example, one woman reported declining endocrine therapy due to resource barriers, consistent with results of prior research reporting that financial concerns are a well-documented reason for nonadherence to endocrine therapy among young women [22]. Contextualizing these narratives within the broader survivorship rhetoric suggests that financial toxicity may be an important mediator between treatment-related adverse events and diminished quality of life and worse clinical outcomes due to treatment nonadherence.

Forgoing rehabilitation or treatments that can reduce the severity of adverse events may perpetuate financial toxicity and contribute to indirect costs. Women in our study frequently recognized diminished job performance, lost work hours, and vocational disruption. These experiences are corroborated by quantitative studies demonstrating that these issues are particularly salient for young women. In their study comparing lost work-productivity due to breast cancer treatment in women aged 18–44 versus 45–64 years using the 2000–2010 National Health Interview Survey, Ekwueme et al. reported that work loss costs were higher per capita among younger employed women [23]. In their multi-national prospective cohort study of young women with breast cancer, Rosenberg et al. showed that 7% of women employed before diagnosis had become unemployed at 1 year and another 7% endorsed diminished productivity in spite of retaining employment [24]. A secondary analysis of data from this cohort showing discrete trajectories for financial difficulty over time suggested that arm morbidity after treatment might be predictive of sustained financial hardship [25]. Taken together, these data advocate for additional investigations to establish the degree to which treatment-related adverse events contribute to financial toxicity in the YA population. Findings would not only have significant implications for altering the conceptual framework of cancer and financial distress [26] but also provide foundational knowledge about which interventions to mitigate financial toxicity might be developed.

The majority of research on adverse treatment events relies on claims data, which is not able to capture the complex interplay between material and indirect costs, psychological response, and coping behaviors that contribute to financial toxicity [27, 28]. Accordingly, and perhaps because of this lack of data, there are few interventions that address financial toxicity in the period after cancer treatment when many of these adverse events are having the most profound impact. In this study, patients expressed both frustration about understanding what resources might be available for financial assistance and supportive services and gratitude when patient coordinators or financial service specialists guided them on these aspects of their care. These data suggest that strategies to mitigate financial toxicity that are being investigated in the acute phase of cancer care when patients are receiving treatment may also be effective for treatment-related adverse events that present in a delayed fashion. Financial navigation describes a structured approach to assessing debts, assets, and needs, assisting with applications to financial assistance, and documenting financial wellbeing over time [29]. While many institutions have some form of financial navigation, challenges to implementation impede efficacy and utilization [30]. Efforts to improve implementation and dissemination of financial navigation pathways are in progress with trials such as the Lessening the Impact of Financial Toxicity (LIFT) trial and Cancer-Related financial hardship through Delivery of proactive financial navigation InTervention (CREDIT) [31, 32]. As with other financial services, navigation may not be helpful if patients are unable to take advantage of the resource. Further, barriers to utilizing resources may differ for YAs compared to older patients and require further investigation.

In young adults who may be inexperienced with health insurance policies, improving health insurance literacy could be crucial in preventing financial toxicity. Investigators from the University of Utah Healthcare and Intermountain Health systems piloted a health insurance education program for adolescents and YAs with cancer and found subsequent reduction in financial toxicity and perceived stress in the intervention arm compared to usual care financial navigation [10]. Patients in our study also discussed direct assistance with costs. Unconditional cash transfers may reduce financial hardship based on preliminary evidence from the Guaranteed Income and Financial Treatment (GIFT) trial where cancer patients with Pennsylvania Medicaid status were provided $1000 per month to assist with their needs [33]. Encouraging results from these trials suggest that embedding similar programming may assist with the financial difficulties experienced as a result of treatment-related adverse events.

We acknowledge several limitations of this study. Participants received their care from a single academic institution, and their perspectives may not be generalizable to other YAs with breast cancer. Although patients with stage I–IV breast cancer were included, risk for financial toxicity and treatment-related adverse events may have varied markedly as a result of differences in therapies received. The data collected did not examine whether long-term financial consequences differed for those with chronic treatment-related morbidity compared to patients whose adverse events resolved. As the original investigation aimed to capture experiences with financial toxicity over time, some women were interviewed more than 2 years from the time of their cancer diagnosis and may have reported circumstances of their care in less detail than if captured during treatment. As this qualitative analysis considered only narratives of young adult breast cancer survivors, it does not expand our understanding of how different patient populations might adopt certain coping behaviors over others or exhibit variability in how they leverage their support systems. Patients were not asked specifically or systematically about the availability of resources to reduce their financial burden. Therefore, our ability to identify barriers such as structural gaps or inequities on which to intervene is restricted.

## Conclusion

Treatment-related adverse events can contribute to financial toxicity after breast cancer through direct and indirect costs. Among young adults, indirect costs can include those that result from vocational disruption. Thus, strategies to reduce the risk of financial toxicity should be included in care pathways to address complications of treatment itself.

## Data Availability

No datasets were generated or analysed during the current study.

## References

[1] Cathcart-Rake EJ, Ruddy KJ, Bleyer A, Johnson RH (2021) Breast cancer in adolescent and young adult women under the age of 40 years. JCO Oncol Pract 17(6):305–313. 10.1200/OP.20.00793 33449828 10.1200/OP.20.00793

[2] Zheng Z, Jemal A, Han X et al (2019) Medical financial hardship among cancer survivors in the United States. Cancer 125:1737–1747 30663039 10.1002/cncr.31913

[3] Xu S, Murtagh S, Han Y et al (2024) Breast cancer incidence among US women aged 20–49 years by race, stage, and hormone receptor status. JAMA Netw Open 7(1):e2353331. 10.1001/jamanetworkopen.2023.53331 38277147 10.1001/jamanetworkopen.2023.53331 PMC10818222

[4] Cohen RA, Zammitti EP (2018) High-deductible health plan enrollment among adults aged 18–64 with employment-based insurance coverage. NCHS Data Brief 317:1–8 30156534

[5] Federal Reserve. *Survey of Consumer Finances* (2019).https://www.tellusapp.com/blog/average-savings-by-age

[6] Salsman JM, Bingen K, Barr RD, Freyer DR (2019) Understanding, measuring, and addressing the financial impact of cancer on adolescents and young adults. Pediatr Blood Cancer 66:e27660 30756484 10.1002/pbc.27660 PMC6777708

[7] Bellizzi KM, Smith A, Schmidt S et al (2012) Positive and negative psychosocial impact of being diagnosed with cancer as an adolescent or young adult. Cancer 118:5155–5162 22415815 10.1002/cncr.27512

[8] Myers SP, Aviki E, Sevilimedu V, Thom B, Gemignani ML. Financial toxicity among women with breast cancer varies by age and race. Annals Surg Oncol. Accepted for publication July 2024. 10.1245/s10434-024-15895-5 PMC12016111 39078600

[9] Khera N, Sugalski J, Krause D et al (2020) Current practices for screening and management of financial distress at NCCN member institutions. J Natl Compr Canc Netw 18:825–831 32634772 10.6004/jnccn.2020.7538

[10] Mann K, Waters AR, Park ER, Perez GK, et al. HIAYA CHAT study protocol: a randomized controlled trial of a health insurance education intervention for newly diagnosed adolescent and young adults cancer patients. Trials. 23: 682 10.1186/s13063-022-06590-5 PMC9388989 35986416

[11] Finkelstein ER, Ha M, Hanwright P et al (2022) A review of American insurance coverage and criteria for conservative management of lymphedema. J Vasc Surg Venous Lymphat Disord 10(4):929–936 35364303 10.1016/j.jvsv.2022.03.008

[12] Lee S, Olvera RG, Shiu-Yee K, Rush LJ, Tarver WL, Blevins T, McAlearney AS, Andersen BL, Paskett ED, Carson WE, Chen JC, Obeng-Gyasi S (2023) Short-term and long-term financial toxicity from breast cancer treatment: a qualitative study. Support Care Cancer 32(1):24. 10.1007/s00520-023-08199-z 38095729 10.1007/s00520-023-08199-z

[13] Williams DR, Lawrence JA, Davis BA, Vu C (2019) Understanding how discrimination can affect health. Health Serv Res 54(S2):13740 10.1111/1475-6773.13222 PMC6864381 31663121

[14] Morris WL, Sinclair S, DePaulo BM (2007) No shelter for singles: the perceived legitimacy of marital status discrimination. Group Process Intergroup Relat 10(4):457–470

[15] Collins C, Williams DR (1999) Segregation and mortality: the deadly effects of racism. Sociol Forum 14(2):495–523

[16] Witte J, Mehlis K, Surmann B, Lingnau R, Damm O, Greiner W, Winkler EC (2019) Methods for measuring financial toxicity after cancer diagnosis and treatment: a systematic review and its implications. Ann Oncol 30(7):1061–1070 31046080 10.1093/annonc/mdz140 PMC6637374

[17] Basch E, Reeve BB, Mitchell SA, Clauser SB, Minasian LM, Dueck AC et al (2014) Development of the national cancer institute’s patient-reported outcomes version of the common terminology criteria for adverse events (PRO-CTCAE). J Natl Cancer Inst 106(9):dju244 25265940 10.1093/jnci/dju244 PMC4200059

[18] Lustberg MB, Kuderer NM, Desai A, Bergerot C, Lyman GH (2023) Mitigating long-term and delated adverse events associated with cancer treatment: implications for survivorship. Nat Rev Clin Oncol. 10.1038/s41571-023-00776-9 37231127 10.1038/s41571-023-00776-9 PMC10211308

[19] Berger AM et al (2015) Cancer-related fatigue, version 2.2015. J Natl Compr Canc Netw 13:1012–1039 26285247 10.6004/jnccn.2015.0122 PMC5499710

[20] Fayanju OM, Mayo TL, Spinks TE, Lee S, Barcenas CH, Smith BD et al (2016) Value-based breast cancer care: a multidisciplinary approach for defining patient-centered outcomes. Ann Surg Oncol 23:2385–2390 26979306 10.1245/s10434-016-5184-5

[21] Mathew A, Benny SJ, Boby JM, Sirohi B (2022) Value-based care in systemic therapy: the way forward. Curr Oncol 29(8):5792–5799 36005194 10.3390/curroncol29080456 PMC9406978

[22] Spencer JC, Reeve BB, Troester MA, Wheeler SB (2020) Factors associated with endocrine therapy non-adherence in breast cancer survivors. Psychooncology 29(4):647–654 32048400 10.1002/pon.5289 PMC7190446

[23] Ekwueme DU, Trogdon JG, Khavjou OA, Guy GP Jr. (2016) Productivity costs associated with breast cancer among survivors aged 18–44 years. Am J Prev Med 50(2):286–294 26775908 10.1016/j.amepre.2015.10.006

[24] Rosenberg SM, Vaz-Luis I, Gong J, Rajagopal PS, Ruddy KJ, Tamimi RM et al (2019) Employment trends in young women following a breast cancer diagnosis. Breast Cancer Res Treat 177(1):207–214 31147983 10.1007/s10549-019-05293-x PMC7265819

[25] Myers SP, Zheng Y, Dibble K, Mittendorf EA, King TA, Ruddy KJ et al (2024) Financial difficulty over time in young adults with breast cancer. JAMA Netw Open 7(11):e2446091 39535790 10.1001/jamanetworkopen.2024.46091 PMC11561695

[26] DQ® Adult Treatment Editorial Board. PDQ financial toxicity and cancer treatment. Bethesda, MD: National Cancer Institute; Updated December 13, 2018. https://www.cancer.gov/about-cancer/managing-care/track-care-costs/financial-toxicity-hp-pdq. Accessed 15 Dec 2018

[27] Wong W, Yim YM, Kim A, Cloutier M, Gauthier-Loiselle M, Gagnon-Sanschagrin P, Guerin A (2018) Assessment of costs associated with adverse events in patients with cancer. PLoS ONE 13(4):e0196007 29652926 10.1371/journal.pone.0196007 PMC5898735

[28] Pike CT, Birnbaum HG, Muehlenbein CE, Pohl GM, Natale RB (2012) Healthcare costs and workloss burden of patients with chemotherapy-associated peripheral neuropathy in breast, ovarian, head and neck, and nonsmall cell lung cancer. Chemother Res Pract 2012:913848. 10.1155/2012/913848 22482054 10.1155/2012/913848 PMC3312207

[29] Yezefski T, Steelquist J, Watabayashi K, Sherman D, Shankaran V (2018) Impact of trained oncology financial navigators on patient out-of-pocket spending. Am J Manag Care 24(5 Suppl):S74-Ss9 29620814

[30] Association of Community Cancer Centers (2016) Trends in cancer programs survey. https://www.accc-cancer.org/surveys/CancerProgramTrends-2016-Overview.asp

[31] Wheeler SB, Biddell CB, Manning ML, Gellin MS, Padilla NR, Spees LP et al (2022) Lessening the impact of financial toxicity (LIFT): a protocol for a multi-site, single-arm trial examining the effect of financial navigation on financial toxicity in adult patients with cancer in a rural and non-rural setting. Trials 23:839 36192802 10.1186/s13063-022-06745-4 PMC9527389

[32] ClinicalTrials.gov: NCT04960787: financial navigation program to improve understanding and management of financial aspects of cancer care for patients and their spouses (CREDIT). https://clinicaltrials.gov/ct2/show/NCT04960787

[33] Doherty M, Heintz J, Leader A, Wittenberg D, Ben-Shalom Y, Jacoby J, Castro A, West S (2023) Guaranteed income and financial treatment trial (GIFT Trial or GIFTT): a 12-month, randomized controlled trial to compare the effectiveness of monthly unconditional cash transfers to treatment as usual in reducing financial toxicity in people with cancer who have low incomes. Front Psychol 18:1179320 10.3389/fpsyg.2023.1179320 PMC10234289 37275728

